# The E3 Ubiquitin Ligase TRIM21 Promotes HBV DNA Polymerase Degradation

**DOI:** 10.3390/v12030346

**Published:** 2020-03-21

**Authors:** Ting Mu, Xiaoqing Zhao, Yanan Zhu, Hongxia Fan, Hua Tang

**Affiliations:** Tianjin Life Science Research Center, Tianjin Key Laboratory of Inflammation Biology, Collaborative Innovation Center of Tianjin for Medical Epigenetics, Department of Pathogen Biology, School of Basic Medical Sciences, Tianjin Medical University, Tianjin 30070, China; muting1209@126.com (T.M.); zxq17606322219@163.com (X.Z.); zhuyanan95@126.com (Y.Z.); fanhongxia2006@126.com (H.F.)

**Keywords:** hepatitis B virus, TRIM21, HBV DNA Pol, interaction, ubiquitination

## Abstract

The tripartite motif (TRIM) protein family is an E3 ubiquitin ligase family. Recent reports have indicated that some TRIM proteins have antiviral functions, especially against retroviruses. However, most studies mainly focus on the relationship between TRIM21 and interferon or other antiviral effectors. The effect of TRIM21 on virus-encoded proteins remains unclear. In this study, we screened candidate interacting proteins of HBV DNA polymerase (Pol) by FLAG affinity purification and mass spectrometry assay and identified TRIM21 as its regulator. We used a coimmunoprecipitation (co-IP) assay to demonstrate that TRIM21 interacted with the TP domain of HBV DNA Pol. In addition, TRIM21 promoted the ubiquitination and degradation of HBV DNA Pol using its RING domain, which has E3 ubiquitin ligase activity. Lys260 and Lys283 of HBV DNA Pol were identified as targets for ubiquitination mediated by TRIM21. Finally, we uncovered that TRIM21 degrades HBV DNA Pol to restrict HBV DNA replication, and its SPRY domain is critical for this activity. Taken together, our results indicate that TRIM21 suppresses HBV DNA replication mainly by promoting the ubiquitination of HBV DNA Pol, which may provide a new potential target for the treatment of HBV.

## 1. Introduction

Hepatitis B virus (HBV) infection is a global health problem, with more than 240 million people with chronic HBV infection worldwide, which is closely related to the development of hepatic fibrosis, cirrhosis and hepatocellular carcinoma [1,2]. Hepatocellular carcinoma is the second most common cancer worldwide, with approximately half of cases being caused by HBV infection [3], which leads to 700 thousand deaths each year [4]. The current clinical use of antiviral drugs, such as nucleoside analogs, can inhibit the replication of HBV and the occurrence of inflammation, thereby diminishing the development of liver cirrhosis and hepatocellular carcinoma and reducing the morbidity and mortality [5]. However, the covalently closed circular DNA (cccDNA) of the HBV genome in the nucleus after HBV infection is a key replication intermediate in the viral life cycle and is persistent, so HBV patients still have a potential risk for developing liver cancer [2,3]. Therefore, it is of great significance to explore the regulatory mechanism of HBV replication to find and develop new drugs or targets for treatment.

HBV is a member of the Hepadnaviridae family, and its genome is 3.2kb, partially double-stranded circular DNA, with four overlapping open reading frames (ORFs): P, S, C and X, which encode HBV DNA polymerase (HBV DNA Pol), envelope protein, precore and core protein and X protein (HBx), respectively [6,7,8]. In addition, a recent study in our laboratory has confirmed that HBV encodes a miRNA that regulates the replication of the virus itself and plays an important role in the HBV life cycle [9]. Among the proteins encoded by HBV, HBV DNA Pol plays an important role in HBV replication and has both polymerase and reverse transcriptase activities.

HBV DNA Pol is composed of three functional domains that include TP, RT, and RNaseH, which are responsible for HBV replication, and a variable spacer region. The TP domain of HBV DNA Pol recognizes the ε components of the HBV pre-genome RNA (pgRNA) and forms a complex with the pgRNA, which plays an important role in the formation of the nucleocapsid and initiates the reverse transcriptional reaction [10]. The RT domain with reverse transcription activity of HBV DNA Pol is critical for reverse transcription of the pgRNA into relaxed circular DNA (rcDNA). In addition, HBV DNA Pol is linked to the 5’ terminus of the HBV genome (−)-strand DNA and promotes the synthesis of the (+)-strand of DNA by its polymerase activity. Moreover, the RNase H domain can degrade the majority of pgRNA in the DNA synthesis process [8,10,11,12], so HBV DNA Pol plays a key role in DNA synthesis and pgRNA packaging. Recent studies have indicated that the reverse transcriptase activity of HBV DNA Pol might stimulate the proapoptotic and proinflammatory response, which is an important trigger of cirrhosis, hepatocellular carcinoma and steatosis caused by chronic hepatitis B [13]. Overall, HBV DNA Pol not only plays an important role in HBV replication but is also closely associated with the development of liver diseases caused by chronic hepatitis B, and antiviral drugs. Nucleoside analogs are directed against the reverse transcriptase activity of HBV DNA Pol, the TP and RNase H domains are potential therapeutic targets in the future [14].

Ubiquitination is one form of posttranslational modification; it refers to the specific modification of the target protein by a series of enzymatic reactions of ubiquitin activator (E1), ubiquitin conjugation (E2) and ubiquitin ligase (E3). Ubiquitination plays a vital role in protein degradation, localization and functions [15,16,17]. The documented evidence suggests that ubiquitination is an important mechanism in organisms and regulates almost all host cell processes, including host–pathogen interactions, and is involved in innate and adaptive immune responses. Most E3 ligases participate in different aspects of immune functions, such as immune-regulatory factors [18,19].

The tripartite motif protein family is a new E3 ligase family; its protein members contain three conserved functional domains, namely, RING, one or two B-box and coiled-coil domains, and a variable C-terminal domain, also known as the RBCC (RING-B-Box-Coiled-coil) family [20,21]. To date, more than 70 different TRIM proteins have been found that play vital roles in cell proliferation, differentiation, apoptosis and the innate immune pathway [22]. Recent studies have found that some members of the TRIM family have antiviral activities. For example, both TRIM14 [23] and TRIM22 [24] can inhibit the replication of hepatitis C virus (HCV). TRIM25 interacts with the RNA virus receptor retinoic acid-inducible gene 1 (RIG-1) to promote the antiviral immune response [25]. TRIM52 suppresses the replication of Japanese encephalitis virus [26]; TRIM31 accelerates the formation of mitochondrial antiviral signaling protein (MAVS) prion-like aggregates by promoting K63-linked polyubiquitination of MAVS, then initiated the natural antiviral signaling pathway [27]; and TRIM56 induces the polyubiquitination of cGAS to affect HSV-1 infection [28]. In addition, studies have reported that some TRIM proteins have antiviral activities against HBV replication, such as TRIM14, a type I interferon (IFN-I) stimulated gene that controls HBV replication by targeting HBx [29]; TRIM22, which inhibits the activity of the HBV core promoter to affect HBV replication [30]; and TRIM25, which can inhibit HBV replication dependent on IL-27 [31].

TRIM21 was first identified as an antibody-binding protein and plays a role as an Fc receptor that recognizes intracellular pathogen-bound antibodies and catalyzes the synthesis of K63 ubiquitin chains to activate the innate immune systems and antiviral reactions during pathogen invasion [32,33]. Studies have shown that TRIM21 promotes the degradation of the intracellular double-stranded DNA sensor DDX41 via K48 chain ubiquitination and decreases the production of type I interferon (IFN) to negatively regulate the innate immune response [34]. TRIM21 is also essential for the interaction between N-myc and STAT interacting protein (Nmi) and interferon inducible protein 35 (IFI35) and promotes the K63-linked ubiquitination of Nmi to inhibit the antiviral activity mediated by the Nmi-IFI35 complex [35]. Moreover, after cells were infected by antibody-opsonized virus, TRIM21 exposed the viral nucleocapsid by accelerating the uncoating of the virus to promote RIG-1, and cGAS recognition of RNA and DNA viruses, which allows a rapid immune response to virus infection [36]. However, whether TRIM21 directly targets the proteins encoded by the virus and induces their ubiquitination to regulate virus replication are largely unknown.

In this study, we screened the interacting proteins of HBV DNA Pol using FLAG affinity purification and mass spectrometry assays and revealed that TRIM21 specifically interacts with the HBV DNA Pol. Furthermore, TRIM21 decreased the stability and protein level of HBV DNA Pol via ubiquitination, thus suppressing HBV DNA replication, which may affect the persistence of HBV infection.

## 2. Materials and Methods

### 2.1. Cell Cultures and Transfections

The human hepatoma cell lines Huh7 and HepG2.2.15 were generous gifts from Zhenghong Yuan’s laboratory at Shanghai Medical School, Fudan University. Huh7 cells were propagated and maintained in Dulbecco’s modified Eagle’s medium (DMEM) and HepG2.2.15 cells were maintained in Minimal Essential Medium (MEM)-α (Gibco, BRL, Grand Island, NY, USA) supplemented with 10% fetal bovine serum (FBS, Gibco), 20 mM HEPES, 100 units/mL penicillin and 100 μg/mL streptomycin. MEM-α was additionally supplemented with 2 mM/L glutamine and 200 μg/mL G418. Cultures were incubated in a cell incubator with 5% CO_2_ at 37 °C. All transfections were performed with LipofectAmine 2000 reagent (Invitrogen, Carlsbad, CA, USA) according to the manufacturer’s recommendations. The amount of transferred DNA for 24-well plates was 1μg; for 6-well plates, 3 μg; and for 100 mL culture flask, 10 μg.

### 2.2. FLAG Affinity Purification

Huh7 cells were transfected with FLAG-HBV DNA Pol as described above. At 36 h post transfection, Huh7 cells were lysed with buffer containing 50 mM Tris HCl at pH7.4, 150 mM NaCl, 1 mM EDTA and 1% TritonX-100 and complete protease inhibitor cocktail (Roche, Basel, Switzerland) at 4 °C for 30 min. After centrifugation, 1mL of the resulting supernatant was incubated with 30 μL FLAG affinity beads (Sigma-Aldrich, London, UK) at 4 °C overnight to precipitate the proteins. Then the protein-affinity beads complexes were washed four times with wash buffer containing 500 mM Tris HCl at pH 7.4 and 150 mM NaCl, and the proteins were eluted two times with peptide eluent, mixed with 4×silver dye buffer containing antioxidant, boiled for 10 min, and then processed for SDS-PAGE according to the instructions for the rapid silver staining kit from Beyotime company (Shanghai, China), the sample was sent for analysis by mass spectrometry.

### 2.3. Coimmunoprecipitation and Glutathione S transferase (GST) pull-down assay

Huh7 cells were transfected with FLAG-HBV DNA Pol and HA-TRIM21 for 36 h and then lysed in co-IP buffer containing 5 mM MgCl_2_, 20 mM imidazole, 300 mM KCl, 1% TritonX-100, 5% glycerol and complete protease inhibitor cocktail (Roche, Basel, Switzerland) at 4 °C for 30 min. After centrifugation, the resulting supernatant was incubated with anti-FLAG antibodies or anti-HA antibodies at 4 °C for 8 h. The protein-antibody complexes were precipitated by incubating the lysates with Bioepitope R protein G/A agarose IP reagent beads (Bioworld Technology, Louis, CA, USA) for 14 h under constant rotation at 4 °C. After incubation, the resin was washed four times with lysis buffer, and proteins were eluted with 2×loading buffer, boiled for 5 min, and processed for Western blotting.

GST pull down was similar to co-IP, and Huh7 cells were transfected with GST-Pol and HA-TRIM21 or HA-ΔSPRY. After 36 h, Huh7 cells were lysed with PBS and 1% Triton X-100 at 4 °C for 30 min. Except for precipitating the proteins with Glutathione beads (Sigma-Aldrich, St. Louis, MO, USA), the other processes are the same as for co-IP.

### 2.4. Western Blot

The transfected cells were lysed in RIPA (Radio Immunoprecipitation Assay) buffer. Proteins were separated by 10% SDS–PAGE, transferred to nitrocellulose membranes, and then detected using the appropriate antibodies. Anti-GAPDH, -TRIM21, -HA tag, -ubiquitin, and -Myc tag antibodies were purchased from Saierbio Biotech (Tianjin, China). Anti-FLAG antibodies were purchased from Abcam (Cambridge, UK). Glyceraldehyde-3-phosphate dehydrogenase (GAPDH) was used as an endogenous normalizer.

### 2.5. Immunofluorescence

Huh7 cells were seeded into Lab-Tek II 8-well chamber slides (CC2 Glass slide, Nunc; Rochester, NY). After 18 h, 500 ng of HA-TRIM21 and FLAG-HBV DNA Pol plasmids were transfected with Lipofectamine 2000 for 36 h. The cells were washed with 1×PBS, fixed with 4% paraformaldehyde in PBS for 30 min at 37 °C, and permeabilized with 0.5% Triton X-100 (*v*/*v*), followed by blocking in PBS containing 10% Donkey serum (Solarbio, Shanghai, China) at room temperature for 2 h. Cells were incubated with anti-HA (1:100) and anti-FLAG antibodies (1:500) diluted in blocking buffer overnight at 4°C. The next day, cells were washed three times with 1×PBS and incubated with the secondary antibodies donkey anti-rabbit and anti-mouse (Invitrogen) diluted in blocking buffer in the dark. Then, cells were mounted with DAPI Fluoromount-G (Southern Biotech, Birmingham, Alabama, USA), and images were captured with an LSM 700 confocal microscope (Zeiss, Thornwood, NY, USA).

### 2.6. Ubiquitination Assay

Huh7 cells grown in 100 mL cell culture flasks were transiently transfected with the plasmids pcDNA3, pTRIM21, pSilencer-NC or pshR-TRIM21 and cotransfected with FLAG-HBV DNA Pol and harvested 36 h post transfection. The transfected cells were treated with 10 μM MG132 for 8 h before harvesting. The cells were lysed in lysis buffer (25 mM Tris-Cl pH 7.4, 150 mM NaCl, 1% NP-40, 10% glycerol, 1 mM DTT, 10 mM NaF, 20 mM sodium vanadate, 8 mM β-glycerophosphate, 50 mM chloroacetamide and protease inhibitor cocktail) at 4 °C for 30 min. After centrifugation, the supernatant was incubated with anti-FLAG antibody and incubated for 10 h at 4 °C. The protein-antibody complexes were precipitated from the lysate with BioepitopeR protein G/A agarose IP reagent beads (Bioworld Technology, Louis, CA, USA) for 12 h at 4 °C. After incubation, the beads were washed with ubiquitination buffer four times. The agarose beads bound by antibody and the associated proteins were separated in 10% SDS-PAGE gels and transferred to a polyvinylidene fluoride (PVDF) membrane (Millipore, Billerica, MA, USA). Immunoblotting was carried out using anti-ubiquitin antibody (Sigma) and the secondary antibody Fab-HRP.

### 2.7. HBV DNA Analysis

Huh7 cells were cotransfected with pHBV1.3 (1 μg), and pcDNA3 (2 μg), pTRIM21 (2 μg), pSilencer-NC (2 μg) or pshR-TRIM21 (2 μg), and HepG2.2.15 cells were transfected with pcDNA3 (3 μg), pTRIM21 (3 μg), pSilencer-NC (3 μg) or pshR-TRIM21 (3 μg) in 6-well plates. Then, 72 h post transfection, HBV DNA from core particles was purified, and transfected cells were washed once with ice-cold PBS and lysed in buffer A containing 10 mM Tris-HCl at pH7.4, 1 mM EDTA, 50 mM NaCl and 1% NP-40. Nuclei were pelleted by centrifugation for 5 min at 10,000g. The supernatant was treated with 0.1 mM MgCl_2_ and 0.1 mg/mL DNase I for 30 min at 37 °C. The reaction was stopped by adding EDTA to a final concentration of 1 mM. The viral core particles were precipitated in 0.8 M NaCl, 8% polyethylene glycol solution at 4°C for 1 h. Core particles were resuspended in buffer C containing 10 mM Tris HCl, 100 mM NaCl, 1 mM EDTA, 1% SDS and 0.5 mg/mL protease K and incubated at 56 °C for 2 h. Viral DNA was isolated from core particles by phenol-chloroform extraction and ethanol precipitation. The DNA pellet was rinsed with 75% ethanol and resuspended in 10 μL TE and quantified by qPCR. The levels of HBV DNA are normalized to genomic β-actin levels.

### 2.8. Southern Blot Assay

The cellular replicative intermediate DNA of HBV was detected by Southern blot analysis. The extracted DNA was separated on a 1% agarose gel in 1×TAE buffer at 100 V for 3 h. Then, the gel was depurinated in 0.25 M HCl for 10 min and balanced three times in 0.4 M NaOH for 15 min. The gel was put into transfer film buffer 20×SSC for 5 min, and transferred onto a nylon membrane (GE Healthcare, Waukesha, WI, USA). The membrane was mixed with 4mL DEPC water, 3 mL 20×SSC, 2 mL 50×Denhardt’s and 1 mL 10% SDS and pre-hybridized for 3 h at 65 °C, then, the membrane was hybridized with HBV probes (5′-AAACGCCGCAGACATCCAG) for 24 h at 42 °C, and chemiluminescence (Thermo Scientific, Massachusetts, USA) was used to detect HBV DNA according to the manufacturer’s instructions.

### 2.9. Statistical Analysis

Statistical significance was determined using a two-tailed unpaired Student’s *t*-test. The data represent the means ± S.D. *P* ≤ 0.05 was considered statistically significant. The graphs represent the average results of three experiments.

### 2.10. Plasmid Construction

The HBV DNA polymerase cDNA were cloned into pcDNA3×FLAG vector between the KpnI and NotI sites, and the corresponding deletion mutants and point mutants were amplified from the full-length HBV DNA Pol by PCR with specific mutant primers. The ubiquitin sequences were amplified from genomic DNA and cloned into pcDNA3 vector. The full-length sequences of human TRIM21 (Tianjin Saier Biotechnology, SRCL13641) cDNA was obtained by RT-PCR and cloned into the pcDNA3 vector with FLAG, Myc or HA tag between the EcoRI and XhoI sites, and the deletion fragments and point mutants were cloned the same as above. The shRNA vector against TRIM21 was constructed by annealing synthesized oligos and ligating them into pSilencer 2.1-U6 neo vector (Ambion, 113P06) between the HindIII and BamHI sites. The HBV replication-competent plasmid cloned in pUC18, pHBV1.3, containing 1.3 copies of the HBV genome that could generate HBV particles, was a generous gift from Zhenghong Yuan’s laboratory at Shanghai Medical School, Fudan University. All the specific primers are shown in Table 1. All constructs were confirmed by DNA sequencing.

### 2.11. RNA Preparation and Reverse Transcription Quantitative PCR (RT-qPCR)

For RNA detection, RNA was isolated from hepatoma cells using TRIzol and was then subjected to cDNA synthesis using Moloney murine leukemia virus (M-MLV) reverse transcriptase (Promega, Madison, WI, USA). Quantitative RT-PCR (RT-qPCR) was performed to detect the relative mRNA levels of HBV DNA Pol and TRIM21. qPCR was performed at 94 °C for 10 min, followed by 40 cycles of 94 °C for 30 s, 50 °C for 30 s and 72 °C for 30 s, in an iQ5 real-time PCR detection system -Bio-Rad A SYBR Premix ExTaq II Kit (Takara, Dalian, China) was used following the manufacturer’s instructions. The relative fold-change of the genes was calculated using the 2^–∆∆Ct^ method. The specific primers are shown in Table 1.

## 3. Results

### 3.1. TRIM21 Interacts with HBV DNA Pol

**Figure 1:**
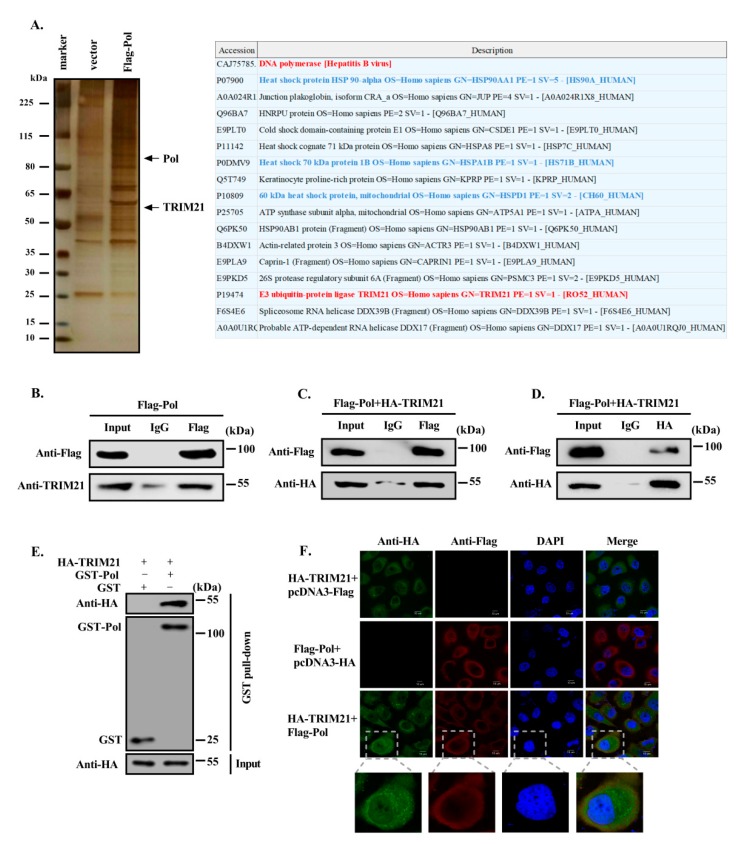
TRIM21 interacts with HBV DNA Pol. (**A**) Huh7 cells were transfected with FLAG-HBV DNA Pol and vector control, and FLAG affinity beads were used to precipitate all proteins that might interact with HBV DNA Pol. The interacting proteins of HBV DNA Pol were screened by mass spectrometry. Some potential interacting partners of HBV DNA Pol are listed. Red marks the target protein, blue marks proteins that have been reported to interact with HBV DNA Pol. Data are representative of two independent experiments. (**B**) The FLAG-HBV DNA Pol expression plasmid was transfected into Huh7 cells. After 36h, a co-IP assay was carried out with anti-FLAG antibody and control IgG antibody. HBV DNA Pol was then detected with anti-FLAG antibody, and anti-TRIM21 antibody was used to detect the endogenous expression of TRIM21 in Huh7 cells by Western blot. (**C**) Huh7 cells were cotransfected with FLAG-HBV DNA Pol and HA-TRIM21, and co-IP was performed as described in (**B**) to detect the expression of HBV DNA Pol and TRIM21. (**D**) Huh7 cells were transfected as described in (**C**), co-IP was performed with anti-HA antibody, and HBV DNA Pol and HA-TRIM21 expression were detected by Western blot. (**E**) GST-HBV DNA Pol and HA-TRIM21 were transfected into Huh7 cells, 36h after transfection, GST beads were used to pull down GST-Pol with its interacting proteins. HBV DNA Pol was detected with anti-GST antibody, and anti-HA antibody was used to detect the expression of TRIM21. (**F**) FLAG-HBV DNA Pol and HA-TRIM21 were transfected into Huh7 cells; 36h after transfection, the mouse anti-FLAG TRITC-conjugated monoclonal antibody and rabbit anti-HA FITC-conjugated polyclonal antibody were used to stain the cells, and the DAPI were used to stain the nuclei. Then, confocal microscopy images were collected and analyzed for the colocalization of HBV DNA Pol and TRIM21. The scale is 10 μm. Data are representative of three independent experiments with three replicates each.

HBV DNA Pol plays an important role in HBV replication. To determine the interacting proteins that regulate the function and metabolism of HBV DNA Pol, we first transfected Huh7 cells with a plasmid expressing HBV DNA Pol fused with a FLAG tag and screened the proteins that may interact with HBV DNA Pol by FLAG affinity purification technology, which we repeated twice. Silver staining showed some specific bands from the HBV DNA Pol-transfected cell lysate. Mass spectrometry analysis showed that the E3 ubiquitin ligase TRIM21 may be one of the candidates interacting proteins of HBV DNA Pol (Figure 1A). To confirm the interaction of TRIM21 with HBV DNA Pol, we transfected the FLAG-HBV DNA Pol expression plasmid into Huh7 cells. An anti-FLAG antibody was used for coimmunoprecipitation (co-IP), and an anti-TRIM21 antibody was used to detect endogenous TRIM21 by Western blotting. TRIM21 was coprecipitated when HBV DNA Pol was precipitated with the anti-FLAG antibody but could not be precipitated by control IgG. Then, we constructed an overexpression plasmid of TRIM21 fused with an HA tag and cotransfected Huh7 cells with the FLAG-HBV DNA Pol expression plasmid. The interaction between these two proteins was determined by co-IP. The results confirmed the endogenous interaction (Figure 1B) and exogenous interactions (Figure 1C) between TRIM21 and HBV DNA Pol. In addition, we used an anti-HA antibody for immunoprecipitate and the anti-FLAG antibody to detect the expression of HBV DNA Pol (Figure 1D), which further verified the interaction between HBV DNA Pol and TRIM21. To eliminate the effect of a nonspecific interaction between TRIM21 and IgG, we constructed a plasmid expressing Pol with a GST tag and found that GST-Pol can precipitate TRIM21 by GST pull down (Figure 1E). To further determine the subcellular colocalization between TRIM21 and HBV DNA Pol, we transiently transfected Huh7 cells with the FLAG-HBV DNA Pol and HA-TRIM21 expression plasmid. Immunofluorescence combined with confocal microscope analysis showed that HBV DNA Pol is mostly localized in the cytoplasm, whereas the TRIM21 is localized both nucleus and cytoplasm. Furthermore, the distribution of HBV DNA Pol and TRIM21 in the cytoplasm of Huh7 cells overlapped to some extent, suggesting that these two proteins may have colocalization in the cytoplasm (Figure 1F).

### 3.2. TRIM21 Interacts with the TP Domain of HBV DNA Pol via its SPRY Domain

**Figure 2:**
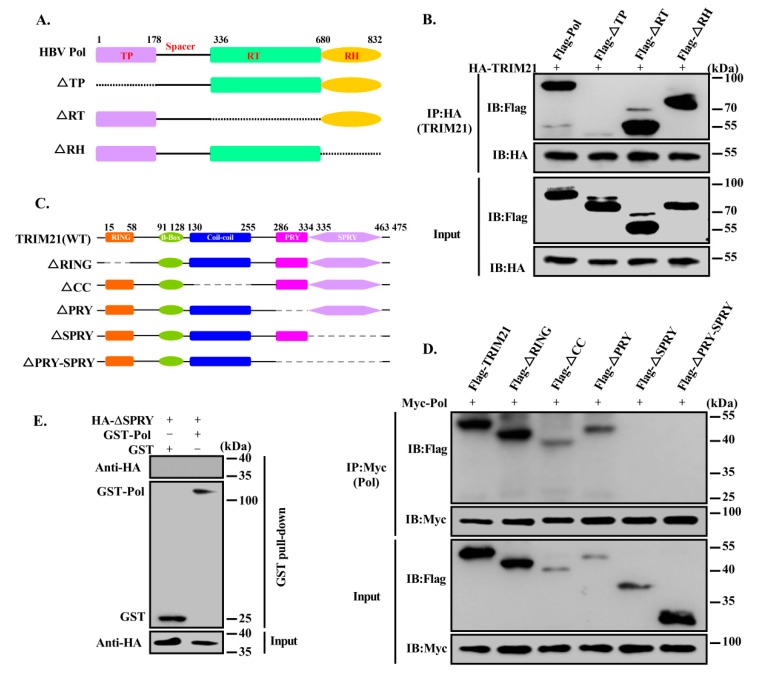
TRIM21 interacts with the TP domain of HBV DNA Pol via its SPRY domain. (**A**) Schematic map of the deletion mutants of HBV DNA Pol. (**B**) HA-TRIM21 was cotransfected with FLAG-HBV DNA Pol, ΔTP, ΔRT or ΔRH. Anti-HA antibody was used for immunoprecipitation, and anti-FLAG antibody was used to detect the expression of HBV DNA Pol. (**C**) TRIM21 and its truncation mutants. (**D**) Huh7 cells were cotransfected with Myc-HBV DNA Pol and FLAG-TRIM21 or its truncation mutants, and co-IP was used to analyze the interaction of Myc-HBV DNA Pol with FLAG-TRIM21 or its truncation mutants. (**E**) GST-HBV DNA Pol and HA-ΔSPRY were transfected into Huh7 cells, and 36 h post transfection, GST beads were used to pull down GST-Pol with its interacting proteins. HBV DNA Pol was detected with anti-GST antibody, and anti-HA antibody was used to detect the expression of ΔSPRY. Data are representative of three independent experiments with three replicates each.

HBV DNA Pol has three functional domains: TP, RT and RNase H domains [37]. To identify which domain is involved in the interaction between HBV DNA Pol and TRIM21, we designed and constructed deletion mutants based on the functional domains (Figure 2A) and performed co-IP analysis to examine their interactions. As shown in Figure 2B, HBV DNA Pol without the TP domain could not interact with TRIM21. Similarly, to determine the TRIM21 domain that interacts with HBV DNA Pol, we deleted either the RING finger, Coiled-coil, PRY or SPRY domain individually or in combination (Figure 2C). The co-IP results showed that TRIM21 lost the ability to interact with HBV DNA Pol when the SPRY domain was deleted (Figure 2D). Furthermore, we used a GST pull-down assay to determine that the SPRY domain of TRIM21 is responsible for the interaction with Pol (Figure 2E). The above results indicate that TRIM21 interacts with the TP domain of HBV DNA Pol via its SPRY domain.

### 3.3. TRIM21 Negatively Regulates the Stability of HBV DNA Pol 

**Figure 3:**
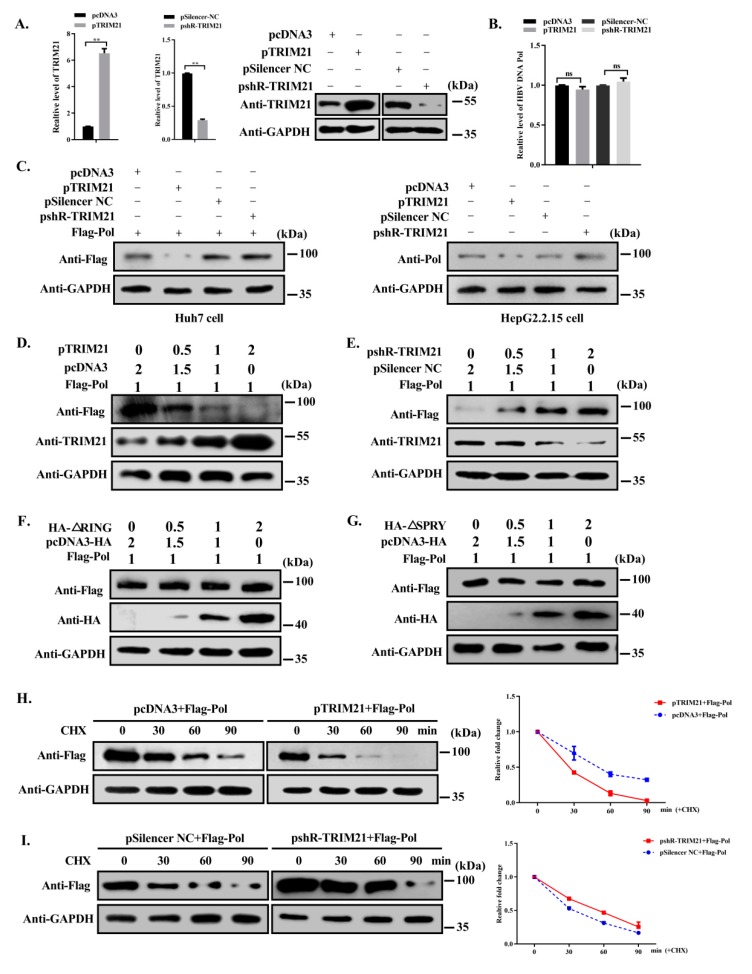
TRIM21 negatively regulates the stability of HBV DNA Pol. (**A**) Huh7 cells were transfected with TRIM21 overexpression or knockdown plasmids or control, and the level of TRIM21 mRNA or protein in the cells was detected by RT-qPCR and Western blot. (**B**) FLAG-HBV DNA Pol was cotransfected with TRIM21 overexpression or knockdown plasmid. The mRNA level of HBV DNA Pol was detected by RT-qPCR. (**C**) Huh7 cells were transfected as described in (**B**), and HepG2.2.15 cells were transfected with TRIM21 overexpression or knockdown plasmids. Western blot was used to detect the protein level of HBV DNA Pol with anti-FLAG antibody or anti-Pol antibody. (**D**) Huh7 cells were transfected with FLAG-HBV DNA Pol along with increasing amounts of TRIM21 expressing plasmid, and WWestern blot was used to detect the protein level of HBV DNA Pol. (**E**) As described in (**C**), except for using pshR-TRIM21 plasmid. (**F**) As described in (**C**), except for using TRIM21-ΔRING plasmid. (**G**) As described in (**C**), except for using TRIM21-ΔSPRY plasmid. (**H**) Huh7 cells were cotransfected with FLAG-HBV DNA Pol and pcDNA3 or TRIM21 plasmid. Thirty hours later, 100μg/ml cycloheximide was used to treat cells, and the protein level of HBV DNA Pol was detected by Western blot. (**I**) Huh7 cells were cotransfected with FLAG-HBV DNA Pol and pSilencer NC or pshR-TRIM21. The treatment was the same as in (**H**). Data are representative of three independent experiments with three replicates each.

Our results above indicated that TRIM21 interacts with HBV DNA Pol. Because TRIM21 is a member of the TRIM family, which has E3 ubiquitin ligase activity, we speculated that TRIM21 may affect the level of HBV DNA Pol. To test this hypothesis, we first verified the efficiency of the constructed expression plasmid of TRIM21 by RT-qPCR and Western blotting (Figure 3A). Next, we examined the effect of TRIM21 on the mRNA and protein level of HBV DNA Pol. As shown in Figure 3B, compared with that in the control, the mRNA level of HBV DNA Pol was not significantly different after overexpression or knockdown of TRIM21. However, the protein level of HBV DNA Pol was significantly decreased or increased when TRIM21 was overexpressed or knocked down, respectively, in Huh7 cells and HepG2.2.15 cells (Figure 3C). In addition, the protein level of HBV DNA Pol decreased gradually with increasing expression of TRIM21 (Figure 3D). In contrast, the protein level of HBV DNA Pol gradually increased with decreasing TRIM21 level (Figure 3E), indicating that the negative regulation of HBV DNA Pol protein levels by TRIM21 was dose-dependent. However, the protein level of HBV DNA Pol was not significantly affected by TRIM21-ΔRING and TRIM21-ΔSPRY mutants (Figure 3F,G).

To examine the effect of TRIM21 on the stability of HBV DNA Pol, we used cycloheximide to inhibit protein synthesis. As shown in Figure 3H, the protein level of HBV DNA Pol decreased gradually with the increasing duration of treatment with cycloheximide, and after overexpression of TRIM21, more than half of the HBV DNA Pol protein in cells treated with cycloheximide for 30 min had been degraded, while the half-life of HBV DNA Pol in the control group was approximately 45 min. On the other hand, the half-life of HBV DNA Pol was longer than that of the control when TRIM21 was knocked down (Figure 3I). These results suggest that TRIM21 shortens the half-life of HBV DNA Pol and decreases the protein level of HBV DNA Pol.

### 3.4. TRIM21 Promotes the Degradation of HBV DNA Pol through the Ubiquitin Proteasome Pathway via Its RING Domain

**Figure 4:**
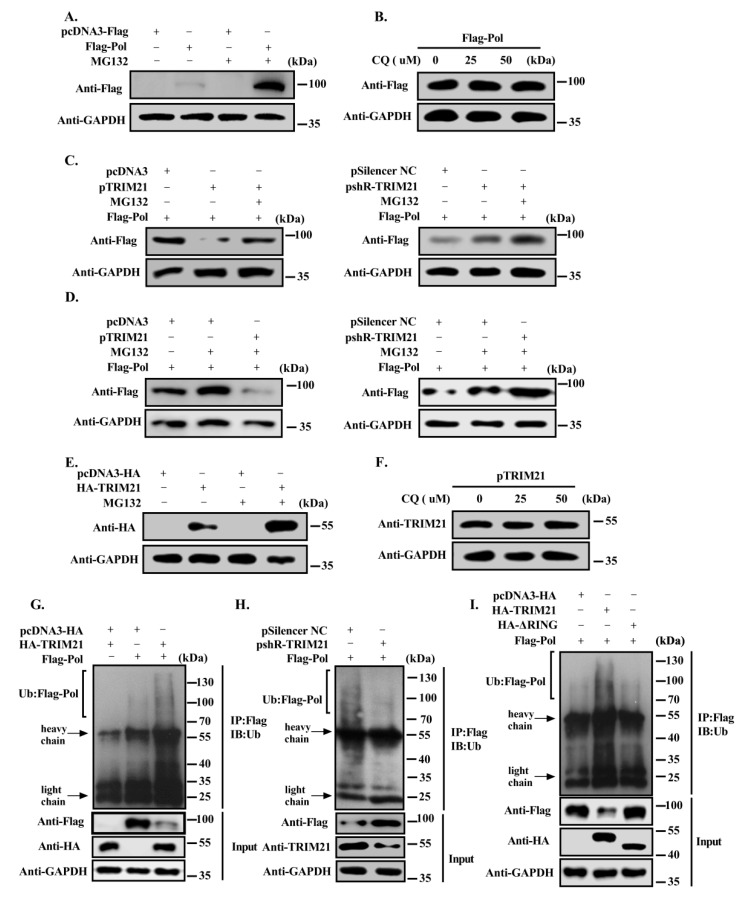
TRIM21 promotes the degradation of HBV DNA Pol through the ubiquitin proteasome pathway via its RING domain. (**A**) Huh7 cells were transfected with FLAG-HBV DNA Pol expression plasmid. After 30 h, the cells were treated with 20 μM MG132 for 8 h, and Western blot was used to detect the protein level of HBV DNA Pol. (**B**) The cells were transfected as described in (**A**) except for using 0, 25 or 50 μM chloroquine to treat the cells for 6h. (**C**) FLAG-HBV DNA Pol was cotransfected with pcDNA3/pTRIM21 (left panel) or pSilencer-NC/shR-TRIM21 (right panel) in Huh7 cells, and MG132 treatment conditions were the same as in (**A**). Then, Western blot was used to detect the protein level of HBV DNA Pol. (**D**) Huh7 cells were cotransfected with FLAG-HBV DNA Pol and pcDNA3 (left panel)/pSilencer-NC (right panel) and treated with MG132 as described in (**A**), or cotransfected with pTRIM21 (left panel)/shR-TRIM21 (right panel). The protein level of HBV DNA Pol was analyzed by Western blot. (**E**) Huh7 cells were transfected with TRIM21 overexpression plasmid, and MG132 treatment was the same as in (**A**). Then, Western blot was used to detect the protein level of TRIM21. (**F**) The same as in (**B**), except for using chloroquine, and the cells were transfected as described in (**E**). (**G**) Huh7 cells were cotransfected with FLAG-HBV DNA Pol and pcDNA3 or HA-TRIM21, and the cells were treated with 10 μM MG132. Subsequently, anti-FLAG antibody was used to precipitate the proteins for ubiquitination analysis. (**H**) The same as in (**G**), except for pSilencer-NC and shR-TRIM21, which were separately cotransfected with FLAG-HBV DNA Pol. (**I**) Huh7 cells were cotransfected FLAG-HBV DNA Pol with wild-type TRIM21, TRIM21-ΔRING or pcDNA3. The ubiquitin antibody was used to detect the ubiquitination level of HBV DNA Pol, and the anti-FLAG antibody was used to detect the protein level. Data are representative of three independent experiments with three replicates each.

There are two main pathways for protein degradation: the lysosome and proteasome pathways. To determine the main degradation pathway of HBV DNA Pol mediated by TRIM21, first, we used the proteasome inhibitor MG132 or the lysosome inhibitor chloroquine to treat HBV DNA Pol-transfected Huh7 cells for 8 h or 6 h, and HBV DNA Pol protein levels were detected by Western blotting. The results showed that HBV DNA Pol levels increased significantly after treatment with MG132 (Figure 4A), but were not obviously changed after chloroquine treatment (Figure 4B), indicating that HBV DNA Pol was mainly degraded by the proteasome pathway. Then, Huh7 cells were cotransfected with FLAG-HBV DNA Pol and TRIM21 overexpression or knockdown plasmid and were treated with MG132 for 8 h before the cells were lysed. The results showed that TRIM21 obviously reduced the protein level of HBV DNA Pol, protein degradation was weakened after MG132 treatment, but the protein level of HBV DNA Pol did not return to the level in the absence of TRIM21 overexpression, which may be due to the fact that MG132 not only inhibited the degradation of HBV DNA Pol but also inhibited the degradation of TRIM21 (Figure 4C, left panel). However, MG132 treatment increased the protein level of HBV DNA Pol when TRIM21 was knocked down (Figure 4C, right panel). To determine the role of TRIM21 in the HBV DNA Pol degradation pathway, first, we transfected Huh7 cells with FLAG-HBV DNA Pol and treated with MG132 for 8 h, or cotransfected with TRIM21 overexpression or knockdown plasmid. As shown in Figure 4D, although MG132 could inhibit the protein degradation of HBV DNA Pol, the protein level of HBV DNA Pol also decreased after overexpression TRIM21 (left panel), while the protein level of HBV DNA Pol increased more after knockdown TRIM21 (right panel). Furthermore, we found that the protein level of TRIM21 was increased in Huh7 cells overexpressing TRIM21 with MG132 treatment but not chloroquine, indicating that TRIM21 was also degraded by the proteasome pathway (Figure 4E,F). These results indicate that TRIM21 promotes the degradation of HBV DNA Pol mainly through the ubiquitin proteasome pathway.

Based on the above results, we speculated that HBV DNA Pol may be the substrate of E3 ligase TRIM21, which can be ubiquitinated and degraded by the 26S proteasome. To address this hypothesis, we cotransfected HBV DNA Pol with pcDNA3/HA-TRIM21 or pSilencer-NC/pshR-TRIM21 into Huh7 cells and analyzed the ubiquitination level of HBV DNA Pol. The ubiquitination assay showed that ubiquitinated HBV DNA Pol increased in cells after overexpression of TRIM21 (Figure 4G), while knocking down TRIM21 decreased the ubiquitination level of HBV DNA Pol (Figure 4H). To eliminate the false positive effect of ubiquitin caused by IgG binding to TRIM21, we used control transfections without HBV DNA Pol plasmid to confirm that the ubiquitination of HBV DNA Pol is dependent on the expression of HBV DNA Pol rather than the autoubiquitination of TRIM21. It has been reported that the members of the TRIM protein family are share three conserved functional domains, of which the RING finger is mainly responsible for the E3 ligase activity of this family of proteins. To determine whether the E3 ligase activity of the RING domain of TRIM21 is responsible for the ubiquitination level of HBV DNA Pol, we constructed a TRIM21-ΔRING mutant and found that the ubiquitination level of HBV DNA Pol in the TRIM21-ΔRING mutant-transfected group was significantly lower than that of the TRIM21-transfected group (Figure 4I). These results demonstrate that TRIM21 mainly regulates the ubiquitination of HBV DNA Pol by the E3 ligase activity of its RING domain.

### 3.5. TRIM21 Mediates the K48-Linked Ubiquitination of HBV DNA Pol at K260 and K283

**Figure 5:**
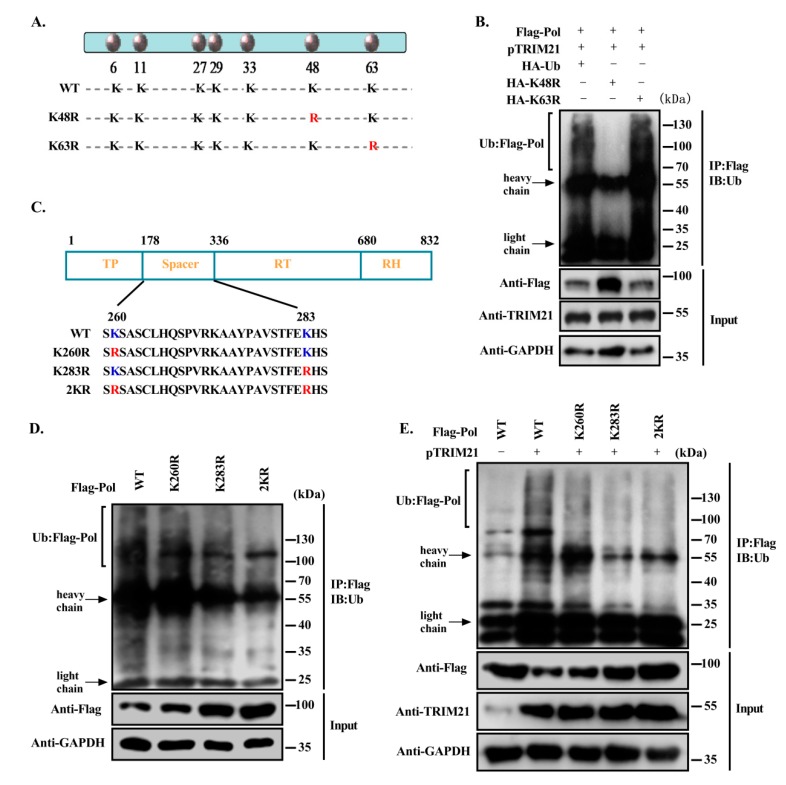
TRIM21 mediates the K48-linked ubiquitination of HBV DNA Pol at K260 and K283. (**A**) A schematic diagram of the lysine point mutations of ubiquitin. (**B**) Overexpression of HBV DNA Pol and TRIM21 in Huh7 cells cotransfected with HA-ubiquitin, Ub-K48R or Ub-K63R mutants. A ubiquitination assay and Western blot were used to detect the ubiquitination and protein level of HBV DNA Pol. (**C**) Schematic of the potential ubiquitination sites of HBV DNA Pol. (**D**) Huh7 cells were transfected with FLAG-HBV DNA Pol or its lysine point mutants, and ubiquitination assays were used to analyze the ubiquitination level of HBV DNA Pol and its lysine mutants. (**E**) TRIM21 and FLAG-HBV DNA Pol or its lysine mutants were cotransfected into Huh7 cells, and the effect of TRIM21 on the ubiquitination level of HBV DNA Pol and its lysine mutants was detected by ubiquitination assay. Data are representative of three independent experiments with three replicates each.

The ubiquitin molecule has seven lysine residues at positions 6, 11, 27, 29, 33, 48 and 63. Most of the current studies focus on Lys48 and Lys63. Lys48-linked polyubiquitin can mediate the recognition of substrate by the proteasome and degradation in the 26S proteasome. Lys63-linked mono/polyubiquitination can alter the function of proteins by affecting cellular localization, DNA repair mechanisms and protein interactions, and can play various other nondegradation-associated roles [16,38]. To characterize the specific type of ubiquitination of HBV DNA Pol by TRIM21, we constructed two ubiquitin point mutants by mutating the lysine at the 48th or 63rd position of ubiquitin to arginine, and the constructs were named K48R and K63R, respectively (Figure 5A). The ubiquitination level of HBV DNA Pol in Huh7 cells transfected with the ubiquitin mutants was detected. The results showed that the ubiquitination level of HBV DNA Pol was significantly decreased in cells expressing K48R, and the intracellular protein level was increased, while K63R expression had no obvious effect on the ubiquitination and protein levels of HBV DNA Pol (Figure 5B). These results suggest that TRIM21 primarily promotes the K48-linked ubiquitination of HBV DNA Pol.

The ubiquitination of proteins mostly occurs on the lysine residues of the proteins. To identify the ubiquitination sites of HBV DNA Pol facilitated by TRIM21, we predicted the ubiquitination sites of HBV DNA Pol with the online programs UbPred (http://www.ubpred.org/) and CKSAAP (http://protein.cau.edu.cn/cksaap_ubsite/), which identified two potential ubiquitination sites at Lys260 and Lys283. Therefore, we constructed point mutants of HBV DNA Pol to replace the lysine residues at positions 260 or 283 with arginine individually or together, and the constructs were named K260R, K283R or 2KR, respectively (Figure 5C). The ubiquitination assay results showed that the ubquitination levels of the point mutants of HBV DNA Pol were less than those of wild-type HBV DNA Pol (Figure 5D), and TRIM21 also had a weaker effect on the ubiquitination of these HBV DNA Pol mutants compared with that of wild-type HBV DNA Pol (Figure 5E), indicating that TRIM21 promoted the ubiquitination of HBV DNA Pol at the Lys260 and Lys283 sites.

### 3.6. TRIM21 Restricts HBV DNA Replication, and the SPRY Domain is Crucial for the Antiviral Role of TRIM21

**Figure 6:**
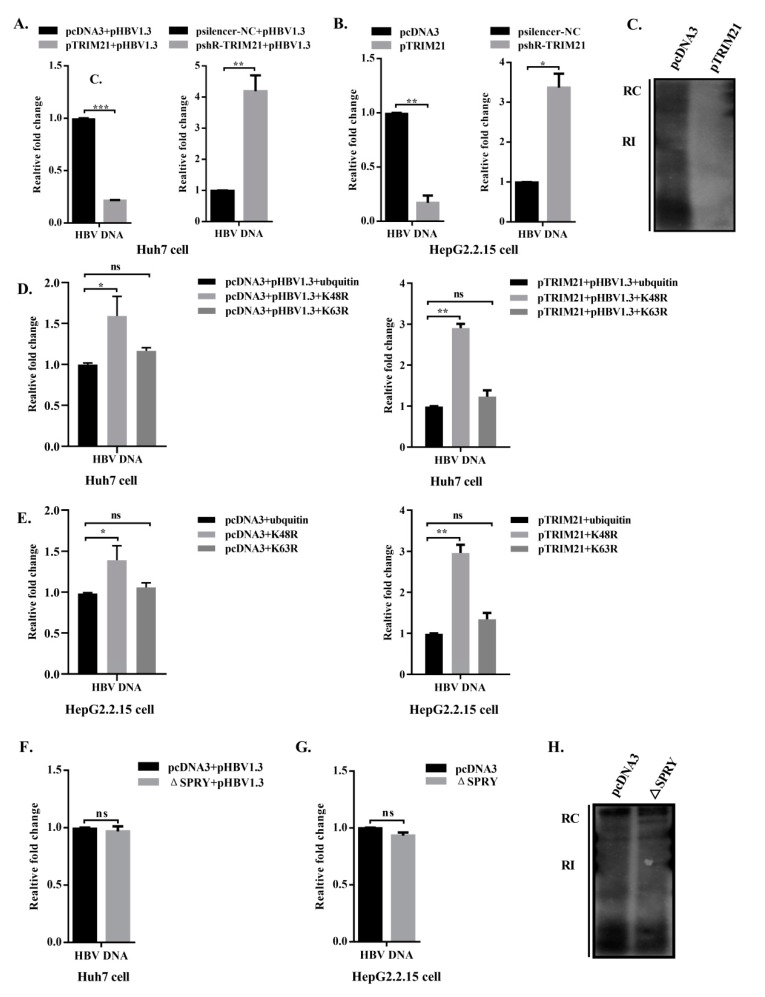
TRIM21 restricts HBV DNA replication. (**A**) Huh7 cells were cotransfected with pHBV1.3 and pcDNA3 or TRIM21 plasmid or pSilencer-NC or shR-TRIM21. qPCR was used to detect HBV DNA. (**B**) HepG2.2.15 cells were transfected with pcDNA3, TRIM21, pSilencer-NC or shR-TRIM21. HBV DNA was detected as described in (**A**). (**C**) HepG2.2.15 cells were transfected with pcDNA3 or TRIM21, and Southern blot was used to detect the cellular replicative intermediate DNA (RI-DNA). (**D**) Huh7 cells were transfected with pHBV1.3, pcDNA3 (left panel) or pTRIM21 (right panel) and ubiquitin or K48R or K63R. HBV DNA was detected by qPCR. (**E**) Except for pHBV1.3, HepG2.2.15 cells were transfected as described in (**D**), and the relative levels of HBV DNA were detected in HepG2.2.15 cells. (**F**) Huh7 cells were cotransfected with pHBV1.3 and pcDNA3 or TRIM21-ΔSPRY, and the HBV DNA level was detected by qPCR. (**G**) Except for pHBV1.3, HepG2.2.15 cells were transfected as described in (**F**). (**H**) HepG2.2.15 cells were transfected with pcDNA3 or TRIM21-ΔSPRY, and RI-DNA was examined by Southern blot. Data are representative of three independent experiments with three replicates each. The data represent the means ± S.D. * *P* < 0.05, ** *P* < 0.01, *** *P* < 0.001.

HBV DNA Pol plays important role in the HBV replication cycle, including viral RNA binding, RNA packaging, protein priming, template switching, DNA synthesis and RNA degradation [14]. However, decreasing HBV DNA Pol obviously reduces the level of HBV DNA in Huh7 cells transfected pHBV1.3 and HepG2.2.15 cells integrated HBV dimer DNA. Therefore, we detected the effect of TRIM21 on HBV DNA by qPCR and on the cellular replicative intermediate DNA (RI-DNA) by Southern blot. The results showed that the levels of HBV DNA were significantly decreased after overexpression of TRIM21 in Huh7 cells, while the expression of HBV DNA in knockdown cells was higher than that in the control group (Figure 6A). Similar results were obtained in HepG2.2.15 cells (Figure 6B). The Southern blot assay showed that overexpression of TRIM21 obviously reduced the level of RI-DNA in the HBV genome (Figure 6C). These results suggest that TRIM21 decreases the levels of HBV DNA and RI-DNA. 

To determine whether the decrease in HBV DNA levels by TRIM21 is supported by the K48-linked ubiquitination of HBV DNA Pol, we examined the change of HBV DNA upon overexpression of wild-type ubiquitin, K48R or K63R when TRIM21 in presence or absence. In the overexpressed ubiquitin group, the level of HBV DNA increased after overexpression of K48R, and it increased more significantly when TRIM21 was overexpressed, suggesting that TRIM21 increased ubiquitin K48R mutant conjugation to prevent endogenous ubiquitin WT (Wild Type) conjugation from HBV DNA Pol degradation, but there was no significant difference in the K63R group whether TRIM21 was overexpressed or not (Figure 6D). A similar result was obtained in HepG2.2.15 cells (Figure 6E).

Studies have shown that some members of the TRIM family depend on their SPRY or PRY domain to participate in protein interactions and to play an antiviral role [23,39]. To determine whether the restriction of HBV DNA by TRIM21 depends on its SPRY domain, we cotransfected pHBV1.3 with pcDNA3 or TRIM21-ΔSPRY plasmids into Huh7 cells, detected the level of HBV DNA by qPCR, and detected RI-DNA by Southern blot assay. Compared with control group, TRIM21-ΔSPRY did not significantly affect the production of HBV DNA (Figure 6F). The same results were obtained in HepG2.2.15 cells (Figure 6G). The Southern blot results showed that the RI-DNA level in the TRIM21-ΔSPRY group also showed no significant difference compared with that in the control group (Figure 6H). These results indicate that TRIM21 mainly depends on the SPRY domain to exert its restriction on HBV DNA.

## 4. Discussion

HBV infection causes serious liver diseases, including liver fibrosis, cirrhosis and hepatocellular carcinoma, which have a high mortality rate [40]. Elevated HBV DNA levels have been shown to be a powerful predictor of increased risk of liver fibrosis and liver cancer [41,42]. HBV DNA Pol plays important roles at different stages of viral replication, including initiation of nucleocapsid packaging via interaction with viral pgRNA and DNA synthesis [43,44]. These functions suggest that each step or protein associated with HBV DNA Pol in the process of HBV replication may be a potential target for antiviral therapy. To study the related proteins that affect the functions of HBV DNA Pol, we used FLAG affinity purification combined with mass spectrometry to screen proteins that might interact with HBV DNA Pol, and obtained more than 100 potential interacting proteins. Among them, HSP60, HSP70 and HSP90 are proteins known to interact with HBV DNA Pol [45,46,47], which demonstrates the reliability of our data.

Our data showed that the turnover of HBV DNA Pol is fast and the half-life is short, so we speculated that it might be attributed to its ubiquitination modification. Ubiquitination is a common modification after protein translation that can mediate many biological processes, such as protein-protein interaction and cell signaling pathways depending on the type of ubiquitin conjugate [48,49]. In addition, most members of the virus family are also degraded by the ubiquitin proteasome pathway, such as the ubiquitination-mediated degradation of cowpox virus proteins, which provide the diverse roles of ubiquitin in the process of virus replication [50]. The NS1 protein encoded by dengue virus is degraded by polyubiquitination with a K48-linked polyubiquitin chain, which may affect virus replication [51]. The degradation of HBV core protein (HBc) is mediated by NIRF (Np95/ICBP90-like RING finger protein) via the ubiquitin proteasome pathway, which affects the release of virus particles [52]. Previous studies have shown that cIAP2 or c-Abl can induce HBV DNA Pol degradation through the ubiquitin proteasome pathway and subsequently inhibit the replication of HBV [53,54]. In our mass spectrometry results, TRIM21, an E3 ubiquitin ligase, was selected for further study. First, we confirmed the interaction and the possible colocalization between TRIM21 and HBV DNA Pol by co-IP assay and immunofluorescence analysis.

TRIM21, as a member of the TRIM family, is composed of five domains: RING, B-box, coiled-coil, PRY and SPRY. Among them, the RING finger domain is responsible for the activity of the E3 ligase; the B-box and coiled-coil domains mediate the oligomerization of TRIM proteins with other proteins and promote the formation of macromolecular complexes [55]. Differences in TRIM family members usually occur at the C terminus, leading to functional diversity [56]. The most common C-terminal structures are PRY and SPRY, which are involved in protein-protein interactions. For example, TRIM21 binds to IgG Fc with high affinity through the PRY-SPRY domain [57]. TRIM21 also interacts with DDX41 via its PRY-SPRY domains and catalyzes DDX41 ubiquitination-mediated degradation [34,58]. Das A et al. found that TRIM21 can interact with Nmi via its SPRY domain, which controls the innate antiviral response mediated by the Nmi-IFI35 complex [35]. Consistent with these observations, we found that the SPRY domain is responsible for the interaction between TRIM21 and HBV DNA Pol, and the TP domains of Pol interacted with TRIM21. Then, we determined that TRIM21 promotes the degradation of HBV DNA Pol by the ubiquitin proteasome pathway by the E3 ligase activity of its RING domain. At present, most of the studies on the interaction between TRIM21 and viral replication focus on the effect of TRIM21 on the regulation of related signaling molecules in the immune pathway. Here, we propose that TRIM21 directly interacts with the viral-related proteins to affect its functions.

Although a previous study showed that HBV DNA Pol is ubiquitinated by cIAP2 or c-Abl, the domain of Pol interacting with other proteins and the potential lysine sites of ubiquitination were not identified. Protein ubiquitin modification usually occurs on lysine residues. To further identify the ubiquitin sites of HBV DNA Pol catalyzed by TRIM21, we used online tools to predict the potential sites of HBV DNA Pol ubiquitination, and K260 and K283 in the spacer domain of HBV DNA Pol were identified as the lysine sites of HBV DNA Pol ubiquitination by TRIM21 by point mutations and ubiquitination assay. The spacer domain of HBV DNA Pol has been thought to be just a linker between the TP and RT domains [59,60]. However, we found that the ubiquitination sites in the spacer region of HBV DNA Pol play an important role in the stability of HBV DNA Pol.

During the HBV lifecycle, binding of HBV DNA Pol to pgRNA and packaging into viral nucleocapsid to initiate reverse transcription is a key step in DNA replication [61,62,63]. The newly formed nucleocapsid can be encapsulated by the envelope protein to form new intact viral particles that can be released from the cell or can reenter into the nucleus for a new cycle of replication as a template for viral transcripts and pregenome [7]. Based on the ubiquitination regulation of HBV DNA Pol by TRIM21, the protein expression of HBV DNA Pol is reduced, but in transiently transfected Huh7 cells or HepG2.2.15 cells only the level of HBV DNA is reduced. Therefore, we further studied the effect of TRIM21 on HBV DNA replication. The results revealed that TRIM21 overexpression led to the decrease in HBV DNA levels. Moreover, Liu et al reported that the RT/RNase H structure of HBV DNA Pol interacted with interferon gene stimulating protein (STING), which destroyed STING via K63-linked polyubiquitination to inhibit the production of interferon and provided a new mechanism for HBV to escape innate immunity [64]. We found that TRIM21 mediated K48-linked polyubiquitination of HBV DNA Pol to result in its degradation in the ubiquitin proteasome pathway. Some studies have shown that the expression of TRIM21 is induced by type I and type II interferon (IFN) via their regulatory factor IRFs [65]. Thus, TRIM21 may have other mechanisms to affect the level of HBV DNA or viral replication. This is the first study to show that TRIM21 restrict HBV DNA replication by binding to and regulating viral proteins rather than the immune system. However, whether HBV DNA Pol has an effect on the expression of TRIM21 and the antiviral function of TRIM21 on HBV may also use the immune pathway requires further study.

**Figure 7:**
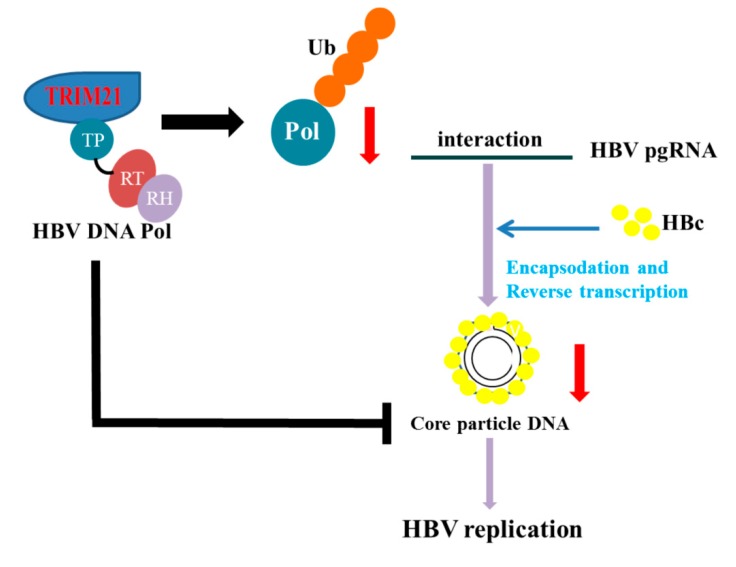
A proposed model describing the role of TRIM21 in the regulation of HBV DNA Pol and HBV DNA replication. HBV-encoded DNA Pol forms a complex with pgRNA and is packaged into a new viral particle by core protein. Some particles reenter into the nucleus and others are enveloped and released from cells for reinfection. Thus, HBV DNA Pol is necessary for HBV replication. Here, we show that TRIM21 mainly promotes the ubiquitination and degradation of HBV DNA Pol, which reduces HBV DNA replication.

In conclusion, we found that TRIM21, an E3 ubiquitin ligase, mainly promoted ubiquitin modification at K260 and K283 of HBV DNA Pol and accelerated the degradation of HBV DNA Pol via the K48-linked ubiquitin proteasome pathway, thus inhibiting HBV DNA replication (Figure 7). It should be noted that TRIM21 restricts HBV DNA replication not only by degradation of HBV DNA Pol, but also other mechanisms which need to be identified in the further. Our findings suggest a new mechanism underlying HBV DNA replication and may provide a potential target for the developing the treatment of HBV infection.

## Figures and Tables

**Table 1:** The primers and oligonucleotides used in this work.

Usage	Primer Name	Sequence (5′–3′)
Construction for HBV DNA Pol	HBV-DNA- Pol-F	GCCGGTACCGCCATGCCCCTATCTTATCCACAC
	HBV-DNA- Pol-R	ATAAGAATGCGGCCGCTCACGGGGGTCTCCATGCGAC
Construction for HBV DNA Pol-mut	HBV-DNA-Pol-K260R-F	CCAGCAGATCCGCCTCCTGCCTCCACCAAT
	HBV-DNA-Pol-K260R-R	GCAGGAGGCGGATCTGCTGGCAAAGTTTGT
	HBV-DNA-Pol-K283R-F	TGAGAGACACTCATCCTCAGGCCATGCAGT
	HBV-DNA-Pol-K283R-R	CTGAGGATGAGTGTCTCTCAAAGGTGGAGA
Construction for HBV DNA Pol- del-mut	HBV-P-del- TP-mut-F	GCCGGTACCATGTCTACAGCATGGGGCAGAATC
	HBV-P-del- RT-mut-F	CTACTGCCTCTCCCTTATCGTCAATCTTCTCGAGCCAGGTCTGTGCCAAGTGTTTG
	HBV-P-del- RT-mut-R	CTGGCTCGAGAAGATTGACGATAAGGGAGAGGCAGTAG
	HBV-P-del- RH-mut-R	ATAAGAATGCGGCCGCTTACCGTTGCCGGGCAACGGGGTA
Construction for pHBV1.3	pHBV1.3	GGGTTTCGCCACCTCTGACT
Construction for TRIM21	TRIM21-F	GCGGAATTCATGGCTTCAGCAGCACGCTTG
	TRIM21-R	GCTCACCTCGAGTAGTCAGTGGATCCTTGTGATC
Construction for shR-TRIM21	shR-TRIM21-F	GATCCGCAGCACGCTTGACAATGATGCTCGAGCATCATTGTCAAGCGTGCTGCTTTTTGA
	shR-TRIM21-R	AGCTTCAAAAAGCAGCACGCTTGACAATGATGCTCGAGCATCATTGTCAAGCGTGCTGCG
Construction for TRIM21 -del-mut	TRIM21-del-RING-F	GCGGAATTCATGCTGCTCAAGAATCTCCGGCCC
	TRIM21-del-CC-F	ACGCCATGGTCCCTCTTGAGGAGGAGCTCATCTCAGAGCTAGATCGAAGGTGC
	TRIM21-del-CC-R	GCACCTTCGATCTAGCTCTGAGATGAGCTCCTCCTCAAGAGGGACCATGGCGT
	TRIM21-del-PRY-F	AAGATGCTGAGGACATGTGCACAGCACTTTCACTCTGGAAAACATTACTGG
	TRIM21-del-PRY-R	CCAGTAATGTTTTCCAGAGTGAAAGTGCTGTGCACATGTCCTCAGCATCTT
	TRIM21-del-SPRY-R	GCTCACCTCGAGTCACTGGGCACCCAGGACCATAGG
	TRIM21-del-PRY-SPRY-R	GCTCACCTCGAGTCACTGTAGGGCCTGGCTCTGCTG
Construction for Ubiquitin	Ubiquitin-F	GACGAATTCATGCAGATCTTCGTGAAAAC
	Ubiquitin-R	GCTCACCTCGAGCTAACCACCTCTCAGACGCAGGAC
Construction for Ubiquitin -mut	Ubiquitin- K48R-F	CTCATCTTTGCAGGCAGGCAGCTGGAAGAT
	Ubiquitin- K48R-R	ATCTTCCAGCTGCCTGCCTGCAAAGATGAG
	Ubiquitin- K63R-R	GCACCTCGAGCTAACCACCTCTCAGACGCAGGACCAGGTGCAGGGTCGACTCCCTCTGGA
RT-qPCR for TRIM21	TRIM21-qRT-F	CAGTCCTGGTTTCAATGATG
	TRIM21-qRT-R	TGGGCCAAATATAAGGAGGC
RT-qPCR for HBV DNA Pol	Pol-qRT-F	CTCTCTTTACGCGGACTCCC
	Pol-qRT-R	TCTTCTAGGGGACCTGCCTC
qPCR for HBV DNA copies	HBV DNA copies -F	TCTTGCCTTACTTTTGGAAG
	HBV DNA copies -R	AGTTCTTCTTCTAGGGGACC
RT-qPCR for β-actin	β-actin-F	CGTGACATTAAGGAGAAGCTG
	β-actin-R	CTAGAAGCATTTGCGGTGGAC
